# The excessive length of first ray as a risk factor for hallux valgus recurrence

**DOI:** 10.1371/journal.pone.0205560

**Published:** 2018-10-10

**Authors:** Xingchen Li, Min Guo, Yuan Zhu, Xiangyang Xu

**Affiliations:** 1 Orthopaedic Department, Ruijin Hospital North, Shanghai Jiaotong University School of Medicine, Shanghai, China; 2 Orthopaedic Department, Anqing Hospital, Anhui University School of Medicine, Anhui, China; 3 Foot and Ankle Center, Ruijin Hospital, Shanghai Jiaotong University School of Medicine, Shanghai, China; Consorci Parc de Salut MAR de Barcelona, SPAIN

## Abstract

**Background:**

It is still unknown whether the excessive length of the first ray is a risk factor for hallux valgus recurrence. The purpose of this study is to clarify the relationship between the excessive length of the first ray and the recurrence of hallux valgus.

**Methods:**

Between 2008 and 2011, a total of 186 feet (left 105, right 81) who underwent chevron osteotomy combined with distal soft tissue procedure in our foot and ankle center were retrospectively reviewed. A postoperative hallux valgus angle(HVA) ≥20° was defined as recurrence. Patients were divided into two groups: recurrence and non-recurrence group. Weight-bearing radiographs were evaluated preoperatively and at the time of last follow-up for both groups. Radiographic parameters including the length of the great toe(P1), the length of the second toe(P2), the length distance between the first and second metatarsal(D), the hallux valgus angle(HVA) were obtained. The excessive length of the first ray(EL) was calculated using the equation of EL = P1-P2-D.

**Results:**

A total of 45 patients (24.2%) had hallux valgus recurrence at the time of last follow-up with a mean follow-up of 83.7 ±12.1 months (range, 66–110). The mean postoperative P1 was 5.06±0.39cm for recurrence group and 4.84±0.34cm for no recurrence group(p<0.001). The mean post operative EL was 5.71±5.01mm for recurrence group and 1.61±4.09mm for no recurrence group(p<0.001). The predictive cutoff value of postoperative P1 and postoperative EL for hallux valgus recurrence was 4.9cm [odds ratio (OR) = 8.67, p = 0.03] and 0.4cm (OR = 6.79, p = 0.001) respectively.

**Conclusions:**

Significant relationships between postoperative P1, postoperative EL and hallux valgus recurrence were identified according to our radiographic results. A postoperative P1>4.9cm and postoperative EL>0.4cm can be risk factors for hallux valgus recurrence. The appreciation of the excessive length of the first ray prior to surgery may help to improve the surgical outcome.

## Introduction

Hallux valgus is among one of the most common deformities around the foot and ankle. Surgical intervention is indicated for failed conservative treatments including nonsteroidal anti-inflammatory medications, shoe modifications and orthotics[1, 2]. However, complications following deformity correction can be as high as 50%[2, 3]. Recurrence is the most common complication after deformity correction. However, the reason for recurrent hallux valgus is often multifactorial. The anatomic characteristics of the patient, shoewear or surgery related factors, all of these factors can lead to recurrence[4].

The protrusion of the first metatarsal has been associated with hallux valgus. Mancuso [5]found that the mean first metatarsal protrusion distance was significantly higher in the hallux valgus population than in the control patients. They concluded that a zero-plus first metatarsal (a long first metatarsal) could be a significant etiologic factor in the development of hallux valgus and should be part of the preoperative evaluation. Excessive length of the hallux has also been associated with the etiology of hallux valgus[6, 7]. Munuera [8]reported a study of 152 radiographs (98 normal feet and 54 hallux valgus feet). They found that the excessive length of the first metatarso-digital segment could be involved in the development of the hallux valgus deformity. When the foot with a long first metatarso-digital segment fit into a narrow toe box or in the push-off phase in gait, pressure from the medial border of the toe box would push the great toe laterally at the level of metatarsophalangeal joint, causing hallux valgus. However, they only discussed about excessive length of the first metatarso-digital segment as the etiology of hallux valgus, as far as we know none of the reported studies discussed about the relationship between the excessive postoperative length of the first ray and hallux valgus recurrence. Theoretically, if the excessive length of the first metatarso-digital segment could lead to hallux valgus, failure to restore its normal length or length ratio could eventually lead to hallux valgus recurrence, though numerous factors could contribute to hallux valgus recurrence.

The objectives of this study are to: 1) define the geometric calculation for the excessive length of the first ray; 2) clarify the relationship between the excessive postoperative length of the first ray and hallux valgus recurrence; 3) determine the predictive cutoff value of the preoperative and postoperative radiographic parameters for hallux valgus recurrence.

## Materials and methods

This study was approved by the ethics committee of Ruijin Hospital, Shanghai Jiaotong University School of Medicine. Informed consent was obtained from all individual participants included in this study. The study was carried out in accordance with the the Declaration of Helsinki. Over the period from January 2008 to December 2011, a total of 186 feet (left 105, right 81) who underwent chevron osteotomy combined with distal soft tissue procedure in our foot and ankle center were included in this retrospective study. Their clinical data was retrospectively reviewed. All procedures were performed by two senior surgeons.

The inclusion criteria for this study were 1) symptomatic hallux valgus deformity with a HVA>20°[9]. 2) failed conservative treatment including non-steroidal anti-inflammatory drugs (NSAIDs), shoe modifications or orthotics for over 6 months. The exclusion criteria included 1) degenerative change of the first metatarsal phalangeal(MTP) joint. 2) instability of the first ray which needed a Lapidus procedure. 3) hindfoot deformities or trauma. 4) an ongoing infection, peripheral vascular diseases or peripheral neuropathy.

### Radiographic assessment

**Fig 1:**
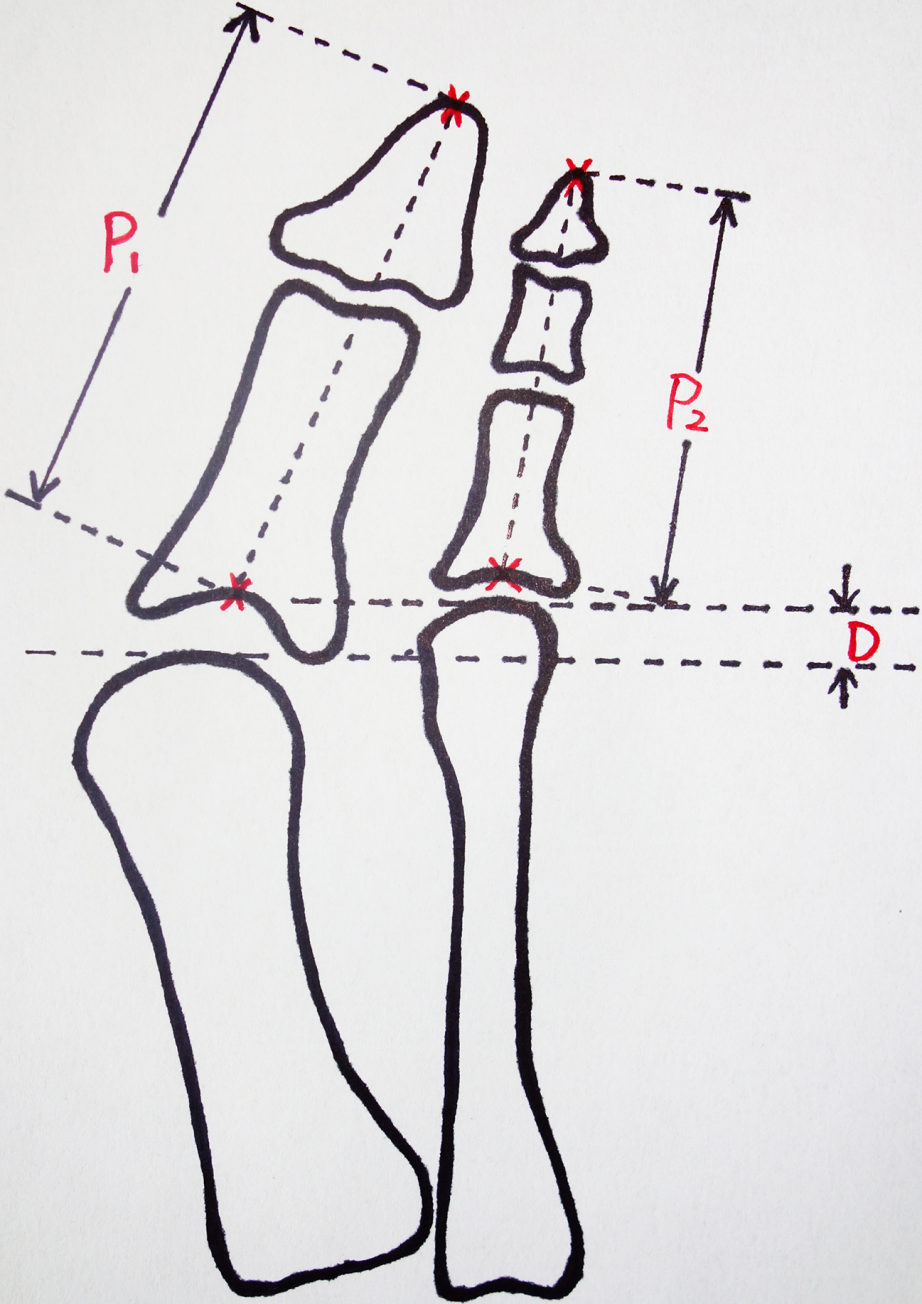
Schematic measurement of the excessive length of the first ray(EL). EL = P1-P2-D. P1, The length of the great toe. P2, The length of the second toe. D, the metatarsal protrusion distance.

The weight-bearing anterioposterior and lateral views of the affected feet were obtained for the evaluation of foot deformity pre-operatively and at the time of last follow-up. HVA was subtended by the lines bisecting the longitudinal axis of the first metatarsal and the proximal phalanx. A postoperative HVA ≥20° were defined as recurrence, with or without symptoms[9–11]. The metatarsal protrusion distance(D) was defined as the distance between 2 transverse lines passing the distal extend of the first and second metatarsals. A positive value was defined as the the second metatarsal longer than the first metatarsal. The length of the great toe(P1) was defined as the distance between the tip of the distal phalanx to the base of the proximal phalanx. The length of the second toe(P2) was defined as the distance between the tip of the distal phalanx to the base of the proximal phalanx. (Fig 1)

The ELwas calculated according to the equation: EL = P1-P2-D. Geometrically, EL was the great toe protrusion distance relative to the second toe in normal aligned individuals. It was defined as the distance between the transverse lines that passing the distal extent of the first and second distal phalanx. However, in case of hallux valgus, as the great toe was displaced laterally and the second toe was often in oblique direction, which made it impossible to measure the correct EL. Thus, we advocated this mathematical equation to calculate EL based on weight-bearing anterioposterior view of the foot.

The radiographs were evaluated by two orthopaedic surgeons (XL, MG). The interobserver reliability was determined by the measurement from both of the two orthopaedic surgeons. XL evaluated all of the radiographs twice at 6 weeks from his previous evaluation to determine the intraobserver reliability. The intraclass correlation coefficients(ICC) and their 95% CI were utilized to determine the interobserver and intraobserver reliability. a value of ICC = 1 was regarded as perfect agreement between the measurements of the two observers, 0.81 to 0.99 indicated excellent agreement, 0.61 to 0.80 indicated good agreement, 0.41 to 0.60 indicated moderate agreement, 0.21 to 0.40 indicated fair agreement, 0.00 to 0.20 indicated poor agreement.

### Statistical methods

The statistical analyses were performed using IBM SPSS Statistics version 23 statistical software. The independent-samples T test was used for the comparison of preoperative and postoperative radiographic parameters between recurrence and no recurrence groups. Receiver operating characteristic(ROC) curve analysis was used to determine the cutoff values for preoperative and postoperative radiographic parameters that could predict hallux valgus recurrence. The cutoff values were determined on the basis of maximal sum of sensitivity and specificity minus 1. Differences with a p value of <0.05 were considered significant.

## Results

A total of 186 feet were included in this retrospective study with an average follow-up duration of 83.7 ±12.1 months (range, 66–110), and an average age of 56.5±11.9 years old (range, 17–84). The average body mass index (BMI) was 23.5±3.0 kg/m^2^ (range, 17.0–32.8). The left foot was involved in 105(56.5%) feet, while the remaining 81(43.5%) feet were on the right side. Akin osteotomy was performed in 124 (66.7%)patients. The Weil osteotomy of the second metatarsal was performed in 68/186 patients (36.6%). A total of 45/186 feet (24.2%) were observed as recurrence by radiographic evaluation at the time of last follow-up. Patients were subdivided into two different groups according to their most recent postoperative HVA, the recurrence group and no recurrence group. The detailed patient demographics for both groups were summarized in Table 1. None of the parameters showed significant difference between these two groups.

**Table 1:** Comparison of patient demographics between recurrence and no recurrence group.

	Recurrence group (N = 45)	No recurrence group (N = 141)	P value
**Age(years)**	58.7±12.2	55.8±11.7	0.15
**BMI(kg/m**^**2**^**)**	22.9±2.9	23.8±3.0	0.10
**Follow-up duration(months)**	81.8±11.9	84.4±12.2	0.22

As shown in Table 2, both of the mean preoperative and postoperative HVA were significantly higher in recurrence group than in no recurrence group. Both of the mean preoperative and postoperative P1 were significantly higher in recurrence group than in no recurrence group. The postoperative EL was significantly higher in recurrence group than in no recurrence group. However, statistic significances were not found for preoperative EL, preoperative P2, postoperative P2, preoperative D, postoperative D between the recurrence and no recurrence groups.

**Table 2:** Comparison of the radiographic parameters between recurrence and no recurrence group.

	Recurrence group	No recurrence group	P Value
**HVA(°)**			
preoperative	39.0±7.7	34.9±7.2	0.001*
postoperative	28.0±6.1	11.2±4.6	<0.001*
**P1(cm)**			
preoperative	5.18±0.34	5.01±0.37	0.039*
postoperative	5.06±0.39	4.84±0.34	<0.001*
**P2(cm)**			
preoperative	4.33±0.66	4.38±0.39	0.556
postoperative	4.32±0.37	4.40±0.34	0.160
**D(cm)**			
preoperative	0.13±0.36	0.14±0.31	0.735
postoperative	0.17±0.38	0.27±0.30	0.058
**EL(cm)**			
preoperative	0.73±0.69	0.53±0.54	0.051
postoperative	0.57±0.50	0.16±0.41	<0.001*

*p value <0.05

**Fig 2:**
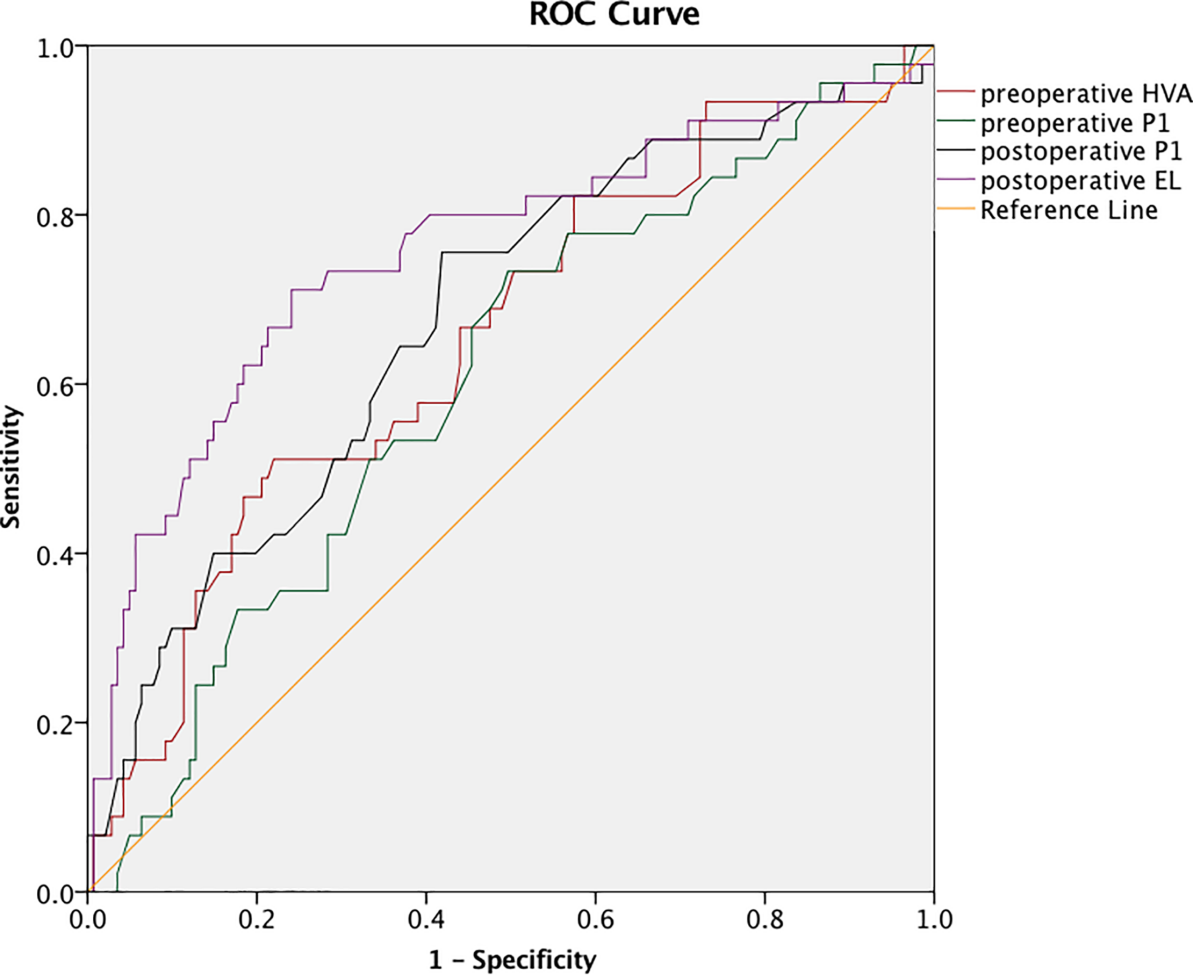
ROC curves. The binary logistic regression analysis was performed to determine the risk factors for recurrence. Significant associations with hallux valgus recurrence were found for preoperative HVA, postoperative P1(OR = 8.67, p = 0.03) and postoperative EL(OR = 6.79, p = 0.001). However, the preoperative P1 (OR = 0.37, p = 0.3) and preoperative EL (OR = 0.8, p = 0.57) did not show significant associations with hallux valgus recurrence. (Table 3).

ROC curve analysis was performed to determine the cutoff values for hallux valgus recurrence. The cutoff values for recurrence were 40.1° for preoperative HVA with the area under curve(AUC) of 0.654, 5.04cm for preoperative P1 with the AUC of 0.607, 4.9cm for postoperative P1 with the AUC of 0.677, 0.5cm for preoperative EL with the AUC of 0.589, 0.4cm postoperative EL with the AUC of 0.757. (Fig 2)

**Table 3:** Association between radiographic parameters and hallux valgus recurrence.

	OR	95%CI	P Value
**HVA** preoperative	1.10	1.83–7.53	0.001*
**P1**			
preoperative	0.37	0.06–2.48	0.30
postoperative	8.67	1.21–61.99	0.03*
**EL**			
preoperative	0.80	0.37–1.72	0.57
postoperative	6.79	2.29–20.08	0.001*

*p value <0.05, OR Odds Ratio, CI confidence interval

The interobserver and intraobserver reliability for two observers (XL, MG) showed good to excellent agreement for all of the radiographs as shown in Table 4.

**Table 4:** Interobserver and intraobserver reliability for measurement of radiographic parameters.

Parameters	Preoperative		Postoperative	
	Interobserver reliability	Intraobserver reliability	Interobserver reliability	Intraobserver reliability
P1	0.903(0.873–0.927)	0.920(0.894–0.939)	0.844(0.797–0.881)	0.891(0.857–0.917)
P2	0.907(0.878–0.930)	0.874(0.836–0.904)	0.834(0.784–0.873)	0.894(0.861–0.92)
D	0.935(0.914–0.951)	0.949(0.933–0.962)	0.934(0.913–0.950)	0.956(0.941–0.967)
EL	0.913(0.886–0.934)	0.884(0.849–0.912)	0.796(0.737–0.843)	0.863(0.821–0.895)

Data were presented as interobserver and intraobserver correlation coefficients with 95% CI

## Discussion

Hallux valgus recurrence was among the most common complications after hallux valgus correction. The recurrence rate following hallux valgus correction vary from 2.7% to 30% according to the literature[9]. As recurrence after hallux valgus correction is directly related to surgical outcome, efforts has been made to identify the risk factors for hallux valgus recurrence. Despite the reason for hallux valgus recurrence was multifactorial[4], radiographic parameters plays a critical role in predicting hallux valgus recurrence.

Okuda[12]clarified the relationship between the hallux valgus angle, intermetatarsal angle and recurrence of hallux valgus. 72 feet were retrospectively evaluated with a mean follow-up of 33 months. Preoperative hallux valgus angle >40° was considered as a risk factor for recurrence(OR = 5.1). In our study, we also found a similar clinical result. Significant association was also found between preoperative HVA and hallux valgus recurrence (OR = 1.1, p<0.001). The preoperative HVA for recurrence group (39.0±7.7°) was significantly higher than no recurrence group (34.9±7.2°). The predictive cutoff value of preoperative HVA for hallux valgus recurrence was 40.1°.

Okuda[11] also reported an incomplete reduction of the sesamoinds can be a risk factor for hallux valgus recurrence. 125 feet were retrospectively assessed with a mean follow-up of 45 months. They found that the feet with postoperative incomplete reduction of the sesamoid had a greater risk of recurrence than those with normal position(OR = 10). DMAA was another important factor for recurrence, failure to correct the distal metatarsal articular surface angle might eventually lead to recurrence[13]. The main problem for the measurement of DMAA was a poor interobserver and intraobserver reliability[14].

**Fig 3:**
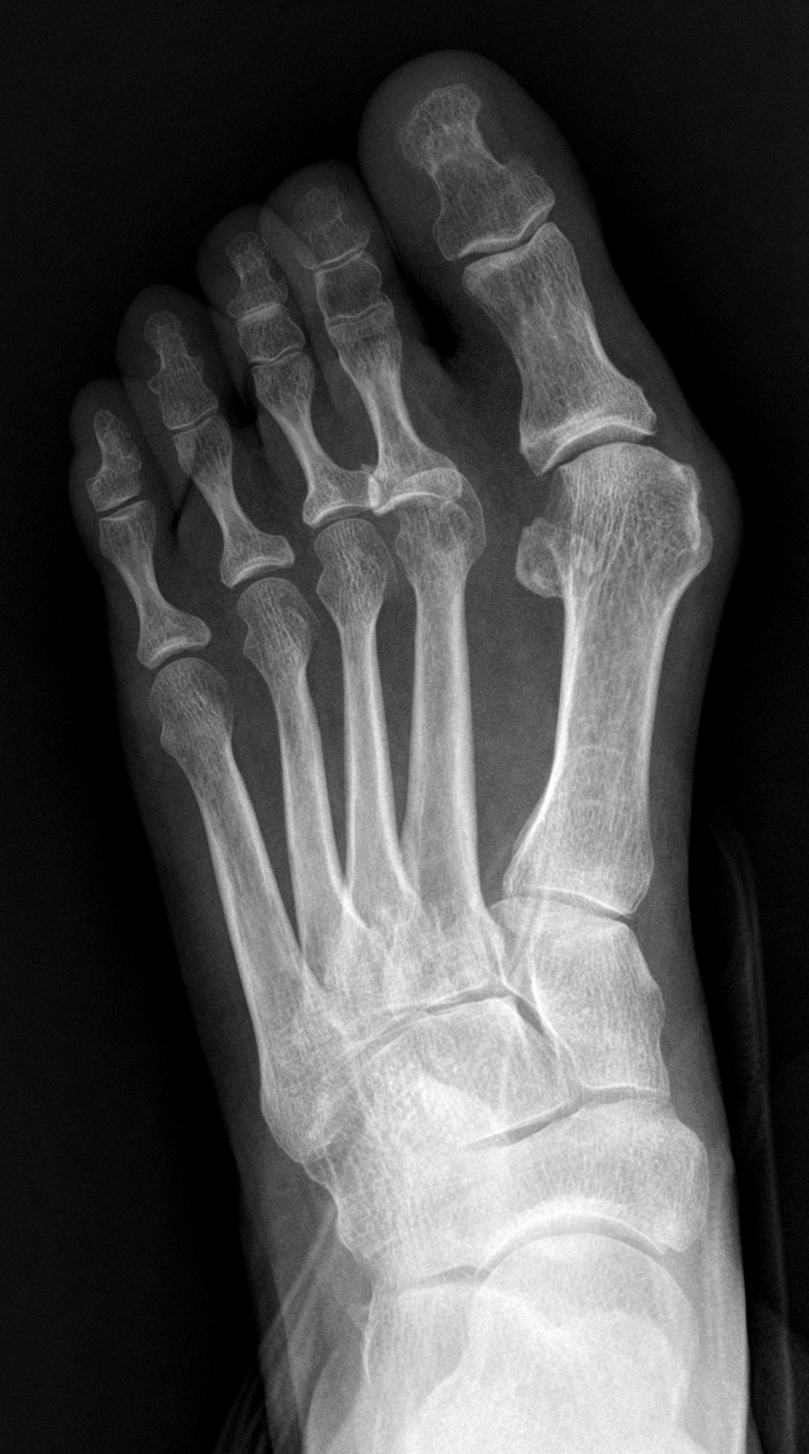
Preoperative weightbearing X-ray of a 55-year-old female. Notice the excessive length of the great toe and protrusion of the first metatarsal.

**Fig 4:**
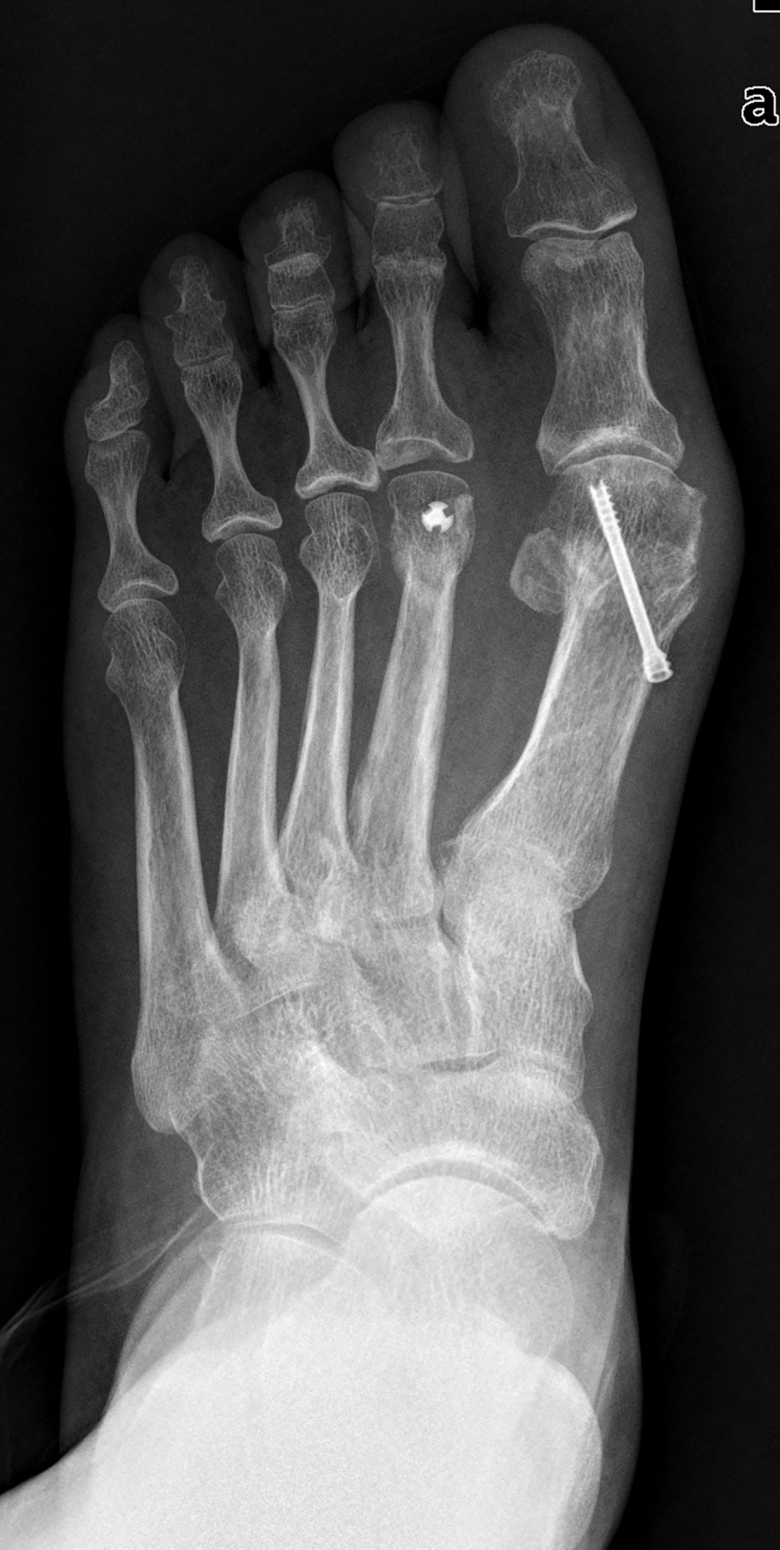
4 months after operation. The excessive length of the first ray had not been addressed.

**Fig 5:**
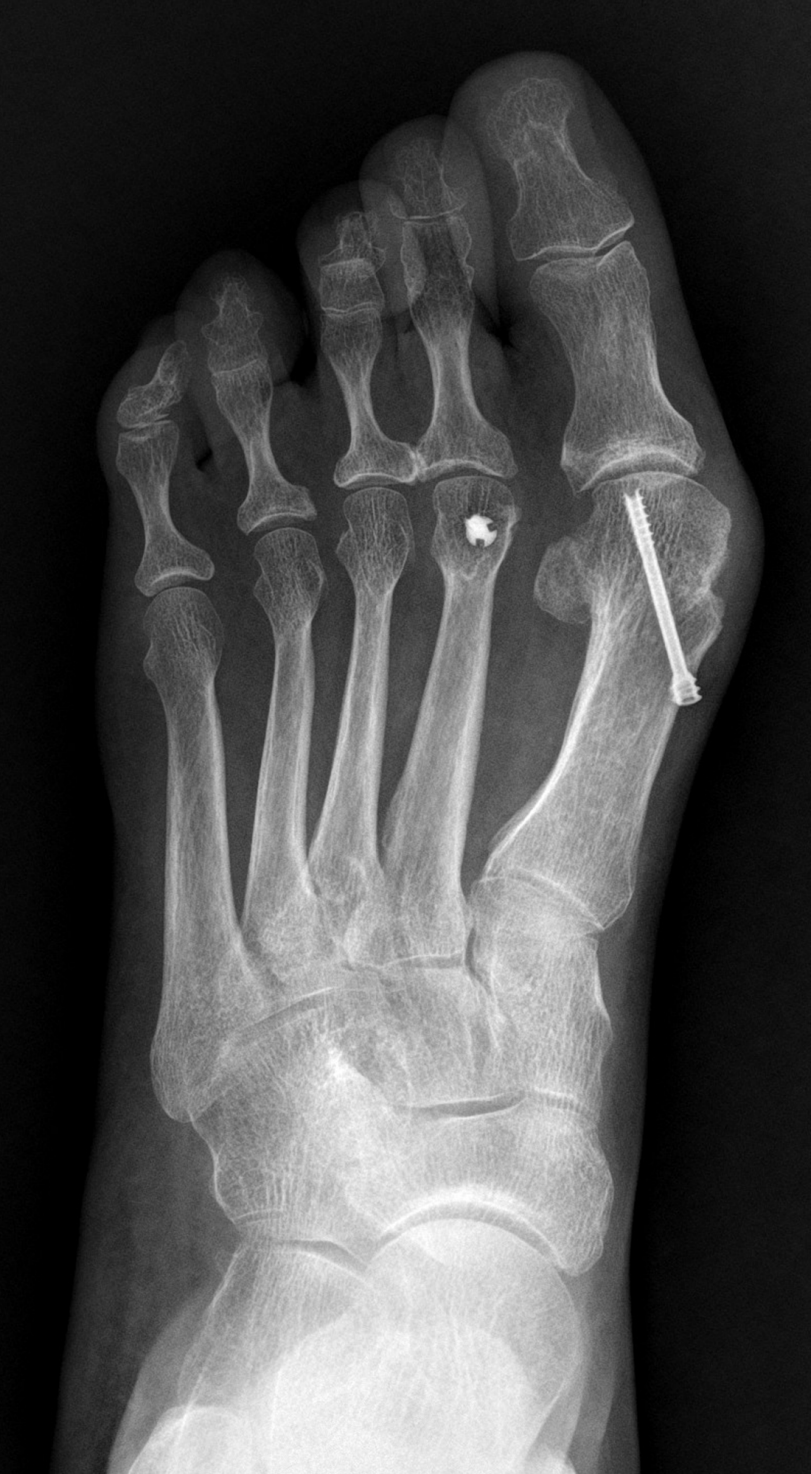
Twenty months after operation. Hallux valgus recurrence was noticed.

In review of the literature, studies had shown wearing shoes with a constricted toe box was associated with hallux valgus[15]. Shoes plays an important role in our life, however foot problems caused by modern footwear has also been recognized. A narrow toe box increases pressures on the medial side of the foot and between toes[16]. Especially for the foot with an excessive length of the first metatarso-digital segment. Failure to restore the normal length of the first ray would eventually lead to an unsuccessful result. In our study, the mean postoperative EL for recurrence group was 0.57±0.50cm, while it was 0.16±0.41cm for no recurrence group(p<0.001). The mean postoperative P1 for recurrence group was 5.06±0.39cm, while it was 4.84±0.34cm for no recurrence group(p<0.001). Significant associations were found between postoperative P1, postoperative EL and hallux valgus recurrence. The predictive cutoff value of postoperative P1 and postoperative EL for hallux valgus recurrence was 4.9cm (OR = 8.67, p = 0.03) and 0.4cm (OR = 6.79, p = 0.001) respectively. However, significant association was not found between preoperative P1(OR = 0.37, p = 0.3), preoperative EL (OR = 0.8, p = 0.57) and hallux valgus recurrence. Though we can not predict hallux valgus recurrence on the basis of these preoperative radiographic parameters, recurrence might be expected if the patient had preoperative excessive length of the first ray and had not been addressed intraoperatively. The patient had preoperative excessive length of the first ray, this analysis will help surgeons with the preoperative plan and counsel the patients about recurrence prior to surgery. (Figs 3–5)

Mathematically, according to the equation EL = P1-P2-D, we can shorten P1 or increase D in order to reduce EL. Shortening of P1 can be achieved by akin osteotomy. While the increase of D is the combination result of Chevron osteotomy of the distal first metatarsal and Weil osteotomy of the second metatarsal. Shortening of the first metatarsal can be achieved by chevron osteotomy, while shortening of the first metatarsal may increase the risk of metatarsalgia after correction[17, 18]. Shortening of the second metatarsal can be achieved by Weil osteotomy[19]. Weil osteotomy of the second toe was indicated when the patients complained about plantar pain and callosity formation below the second metatarsal head. A total of 68/186 patients (36.6%) received the Weil osteotomy of the second metatarsal. and 33/187 patients (17.6%) for the third metatarsal in this study.

Akin osteotomy was a close wedge osteotomy of proximal phalanx of the hallux, which was first described by Akin in 1925. This is a powerful procedure to address enlarged hallux interphalangeal angle. Meanwhile, shortening of the proximal phalanx of the great toe was also noticed after akin osteotomy, it is a result of removal of a bone wedge on the medial side of the proximal phalanx[20]. This is a retrospective study and our surgeons did not shorten the first metatarso-digital segment intentionally during the operation. Akin osteotomy was performed for 124 (66.7%) patients. The difference between postoperative and preoperative P1 for patients underwent akin osteotomy was -0.24cm, while it was -0.11cm for patients without akin osteotomy, significant difference was found between these groups(P = 0.001).

The limitations of this study is its retrospective nature. In this study, we only clarified the relationship between postoperative P1, postoperative EL and hallux valgus recurrence. As all of the subjects in this study were diagnosed with hallux valgus, we did not include normal feet to set the control group. we can only predict hallux valgus recurrence base on the relevant postoperative radiographic parameters. A large sample sized prospective study might be needed to further clarify whether an excessive first metatarso-digital segment can be a risk factor for hallux valgus.

## Conclusions

In conclusion, significant relationship between postoperative P1, postoperative EL and hallux valgus recurrence were found according to our radiographic results. A postoperative P1>4.9cm and postoperative EL>0.4cm can be risk factors for hallux valgus recurrence. The appreciation of the excessive length of the first ray may help surgeons to counsel the patient about outcome prior to surgery the and modifications of the surgical procedures.

## Supporting information

S1 Table Patient database. (XLSX)
